# Variance components for susceptibility to *Mycobacterium bovis* infection in dairy and beef cattle

**DOI:** 10.1186/s12711-014-0077-1

**Published:** 2014-11-18

**Authors:** Ian W Richardson, Dan G Bradley, Isabella M Higgins, Simon J More, Jennifer McClure, Donagh P Berry

**Affiliations:** Smurfit Institute of Genetics, University of Dublin, Trinity College, Dublin, Ireland; Animal and Grassland Research and Innovation Center, Teagasc, Moorepark, Fermoy, Co. Cork Ireland; UCD Centre for Veterinary Epidemiology and Risk Analysis, UCD School of Veterinary Medicine, University College Dublin, Belfield, Dublin 4, Ireland; Irish Cattle Breeding Federation, Bandon, Co. Cork Ireland

## Abstract

**Background:**

Infection of livestock with bovine tuberculosis (bTB; *Mycobacterium bovis*) is of major economical concern in many countries; approximately 15 000 to 20 000 cattle are infected per year in Ireland. The objective of this study was to quantify the genetic variation for bTB susceptibility in Irish dairy and beef cattle.

**Methods:**

A total of 105 914 cow, 56 904 heifer and 21 872 steer single intra-dermal comparative tuberculin test records (i.e., binary trait) collected from the years 2001 to 2010 from dairy and beef herds were included in the analysis. Only animal level data pertaining to periods of herd bTB infection were retained. Variance components for bTB were estimated using animal linear and threshold mixed models and co-variances were estimated using sire linear mixed models.

**Results:**

Using a linear model, the heritability for susceptibility to bTB in the entire dataset was 0.11 and ranged from 0.08 (heifers in dairy herds) to 0.19 (heifers in beef herds) among the sub-populations investigated. Differences in susceptibility to bTB between breeds were clearly evident. Estimates of genetic correlations for bTB susceptibility between animal types (i.e., cows, heifers, steers) were all positive (0.10 to 0.64), yet different from one. Furthermore, genetic correlations for bTB susceptibility between environments that differed in herd prevalence of bTB ranged from 0.06 to 0.86 and were all different from one.

**Conclusions:**

Genetic trends for bTB susceptibility observed in this study suggest a slight increase in genetic susceptibility to bTB in recent years. Since bTB is of economic importance and because all animals are routinely tested at least once annually in Ireland and some other countries, the presence of genetic variation for bTB susceptibility suggests that bTB susceptibility should be included in a national breeding program to halt possible deterioration in genetic susceptibility to bTB infection.

## Introduction

Bovine tuberculosis (bTB) is an infectious chronic respiratory disease caused by *Mycobacterium bovis.* Infection of livestock with bTB has an estimated global cost of €2 billion annually [1], due mostly to the lack of bTB control in developing countries, where infection is endemic, resulting in reduced productivity of livestock. The primary cost of bTB infection in developed countries is the control of bTB, with estimated spends by the Irish and UK governments in 2010 and 2011 of €63 million and €179 million [2], respectively. Bovine tuberculosis is ranked as the fourth most important livestock disease globally [3], primarily due to its importance in developing countries. Eradication of bTB from cattle herds has been successful in some countries such as Australia and throughout Scandinavia. Epidemiological circumstances in developed countries where bTB is present vary. The UK and Ireland have yet to gain an official bTB free status [4], although both countries introduced eradication programs during the late 1950s. In the UK, bTB is an expanding epidemic, while in Ireland, it forms a stable endemic with substantial year-on-year reductions, particularly recently [2]. A compulsory bTB eradication program for cattle was introduced in Ireland in the late 1950s. The initial response was rapid but by the mid-1960s, with the number of animal infections per year in excess of 30 000 [2]. In recent years, the number of animal infections has decreased to less than 20 000 per year; however, progress towards a bTB free status in Ireland remains slow [5,6]. When the eradication program began in the late 1950s, confirmed animal infection prevalence was 17%; in 2003, this prevalence was 0.4% [7]. Re-emergences of bTB have been reported in France and Spain [8-10], with concerns that bTB could expand into neighbouring countries [11].

The existence of wildlife reservoirs for bTB is a major contributing factor to the continued infection of bTB in livestock. In Ireland and the UK, cattle share their environment with wild badger (*Meles meles*) populations in which bTB is endemic [7]. The badger acts as a natural reservoir for the disease [7]. Wildlife reservoirs for bTB in other countries include wild boar and deer in Spain, brush-tailed possums in New Zealand, and white tailed deer in Michigan, USA.

Genetic selection for bTB resistance could be incorporated into current cattle breeding programs in Ireland to enhance the national eradication strategy. However, this approach requires information on the extent of genetic variation for bTB susceptibility within the Irish cattle herd. Heritability for susceptibility to *M. bovis* infection has previously been documented to be 0.18 in both Irish [12] and UK dairy herds [13]. However, these studies were confined to dairy cattle and, to date, no study has been undertaken on beef cattle. Moreover, the potential of genotype by environment (GxE) effects for susceptibility to *M. bovis* infection between herds of different disease prevalence levels has not been quantified.

The objective of the present study was to estimate variance components for bTB susceptibility in both dairy and beef cattle and to quantify whether GxE for bTB susceptibility exists across environments that differ in bTB prevalence. In the present study, tuberculin skin test results from both dairy and beef cattle were used as a measure of bTB susceptibility. The variance components estimated in this study are needed to derive multi-breed estimated breeding values (EBV) for bTB resistance in national genetic evaluations. As well as being useful for incorporation into breeding programs, these EBV could also be useful phenotypes for subsequent genomic analysis.

## Methods

### Testing procedures

Ireland has operated a compulsory bTB eradication program since the late 1950s. The current national program consists of a mandatory registration system for herds, an individual bovine identification system, a computerised movement monitoring system, an animal health computer system, as well as a comprehensive program of disease surveillance and control. Two forms of surveillance are used in Ireland. Field surveillance for bTB is performed annually in each herd through the routine use of the single intra-dermal comparative tuberculin test (SICTT) [14,15]. Testing is carried out by veterinarians approved by the Irish Department of Agriculture, Food and the Marine. In addition, abattoir surveillance is conducted on all animals at slaughter, relying on the gross examination of each carcass by an approved veterinarian for pathology suggestive of bTB.

Field surveillance using the SICTT involves intradermal injection of bovine tuberculin (a purified protein derived from *M. bovis*) into the neck of the animal. This inoculate will cause an immune reaction to the tuberculin in animals that are sensitised to antigens in bovine tuberculin and results in an inflammatory response and localised swelling at the injection site that reaches its greatest intensity 48 to 72 h post-injection. To distinguish animals infected with different strains of *Mycobacterium* from those infected with *M. bovis*, animals are also injected at a different site with avian tuberculin (from *M. avium*). The outcome of the SICTT is determined by the relative difference in the thickness of skin-folds in reaction to the bovine and avian tuberculins. Using the so-called standard interpretation (as would be used in herds with bTB free status that can, therefore, be traded without restrictions), if the relative bovine-avian difference in skin-fold test is greater than 4 mm, the animal is considered a ‘standard reactor’, if between 1 and 4 mm, a ‘standard inconclusive reactor’, and otherwise, a ‘non-reactor’ [15]. Inconclusive reactors are re-tested 60 days after the initial test. Post-mortem examinations and/or laboratory culture of tissue samples can be used to confirm the presence of bTB in reactors.

A herd loses its official bTB free status if at least one reactor is detected; movement of animals from the herd is restricted. Subsequently, infected animals are removed for slaughter and further SICTT testing is conducted at approximately two-month intervals, until two consecutive clear herd tests (i.e., herd tests with no positive or inconclusive reactors disclosed) are achieved, at which point herd bTB-free status is regained. In restricted herds, a lower threshold (so-called, severe interpretation) of the SICTT is used, such that all animals with a bovine-avian difference of at least 2 mm are considered a ‘reactor under severe interpretation’ and animals with a bovine reaction less than 2 mm greater than an avian tuberculin reaction are considered an ‘inconclusive reactor under severe interpretation’. Additional skin tests are always undertaken on herds that are contiguous to, or otherwise linked to, infected herds, and in herds that are located in 'at-risk' areas. Herds are also re-tested for bTB six months after they have re-gained bTB free status.

### Herd-episodes of bTB infection

In the present study, a ‘herd-episode of bTB infection’ refers to the full period of herd restriction triggered by disclosure of bTB infection within a herd [16]. The herd-episode starts with the disclosure or discovery of at least one infected animal (from either field or abattoir surveillance) and ends immediately following two consecutive clear herd tests at approximately two-month intervals. In the present study, herd-episodes were only included in the analysis if at least two standard reactors were detected from field surveillance, of which at least one had to be home-born. Herd-episodes with ten reactors or more were only included if at least 20% of the reactors, based on SICTT results, presented bTB lesions postmortem from abattoir testing.

### Data collection and editing

Tuberculin skin test results from 22 381 herd-episodes in 16 717 dairy, beef and mixed herds were collected between 2001 and 2010, inclusive. Herds were classified as either dairy, beef or mixed, determined by the average breed composition of cows in each herd. Herds were defined as dairy herds if the average breed composition of that herd was ≥90% dairy breed, while herds were defined as beef herds if the average breed composition of that herd was ≥30% beef breed. All other herds were defined as mixed herds. The total number of breeds represented across all herd-episodes was 35, with the mean number of breeds per herd-episode being 2.3; 5571 herd-episodes had more than one breed present during a herd-episode, across both dairy and beef herds.

Only positive and inconclusive results were recorded in the database. During these herd-episodes, there were a total of 183 955 ‘standard reactors’ and ‘reactors under severe interpretation’, 20 884 ‘standard inconclusive reactors’, and 1896 ‘inconclusive reactors under severe interpretation’. Confirmed bTB lesions were identified in 50 614 reactors from postmortem examinations and/or tissue culture samples. The national animal identification and movement system, which monitors animal movements in and out of herds, was used to identify animals present in herds at the time of testing. In total, 1 357 791 animals were present during herd-episodes but did not test positive or inconclusive for bTB. Animals were only included if they had moved into a herd more than six weeks prior to the start of a herd-episode. In the present study, only ‘standard reactors’ and ‘reactors under severe interpretation’ were considered to be infected with bTB; thus the inconclusive reactors were removed from the dataset. Animals with no recorded sire were discarded.

Animal age at the start of each herd-episode was determined. Female animals that were more than 30 months old or had calved at least once by the start of the herd-episode were classified as cows, and otherwise as heifers. Male animals that were less than 36 months at the start of the herd-episode were classified as steers (i.e., castrated bulls), and otherwise as bulls. Due to the paucity of data (n = 1822), bulls were subsequently discarded from the dataset. The final dataset consisted of 21 872 steers, 105 914 cows and 56 904 heifers; of these, 22 573 were reactors and 162 117 were nonreactors.

### Heterosis and recombination effects

Considerable cross-breeding exists in Irish herds. For example, 58% of herd-episodes in beef herds contained animals with direct dairy ancestry. Many cows in beef herds are first or second crosses from dairy herds. Following VanRaden and Sanders [17], heterosis was calculated as $1-{\displaystyle {\sum}_{i=\kern0.5em 1}^n sir{e}_i}\cdot da{m}_i$, and recombination loss as $1-{\displaystyle {\sum}_{i=\kern0.5em 1}^n\left( sir{e}_i^2+da{m}_i^2\right)/2}$, where *sire*_*i*_ and *dam*_*i*_ are the proportion of breed *i* in the sire and dam, respectively.

### Data analysis

Variance components for bTB susceptibility, as a binary trait (i.e. reactor or not reactor), were estimated using both an animal linear mixed model and a threshold animal model in ASreml [18]. Variance components were estimated separately for cows, heifers, and steers, as well as within beef, dairy and all herds; the latter also included mixed herds. Fixed effects considered for inclusion in all models were herd-episode and both heterosis and recombination loss coefficients of the animal (as continuous variables). Parity (1, 2, 3, 4, ≥5) and stage of lactation (0–60, 61–120, 121-180, 181–240, 241–300, >300 days) were included as fixed effects in the analysis of cows. Age (in days) was included as a covariate in the analysis of steers and heifers. Animal sex was included in models for the combined analysis of cows, heifers, and steers. Animal was included in all models as a random genetic effect and founder animals were allocated to genetic groups by breed; preliminary analysis revealed no permanent environmental effects and therefore was ignored in the analyses.

Genetic correlations for bTB susceptibility between animal type (i.e., cows, heifers, and steers) were estimated using bivariate sire models to evaluate whether bTB was genetically the same trait in the different populations. To test whether the genetic correlations between animal types differed from one, using the linear model, a likelihood ratio test was performed between each model and an analysis where the genetic correlation between animal types was constrained to be one. In addition, using the linear model, a likelihood ratio test was performed between each model and a model where the genetic variances in each animal type were constrained to be equal.

Sire estimated breeding values (EBV) for bTB susceptibility were generated from the univariate animal model using only cow records. The mean EBV per breed was obtained for (1) all sires with daughter records for bTB in the data and (2) all sires with at least ten daughter records for bTB in the data. Mean EBV per year of birth was calculated to quantify genetic trends. Only sires born after 1992 were included to calculate the mean EBV per breed and genetic trends, because animals born before 1992 were too distant from the actual phenotypic data. The prevalence of bTB infection in the daughters of sires was calculated as the ratio of non-infected to infected daughters present during a herd-episode. Only sires with more than 50 daughters in more than ten herds were included in this analysis.

Herd-episodes were split into four categories based on mean bTB prevalence to define different environments: very low (<0.25 prevalence), low (0.25 to 0.50 prevalence), high (0.50 to 0.75 prevalence) and very high (>0.75 prevalence). (Co) variance components were estimated within each environment using both linear and threshold animal models. For comparative purposes, variance components estimated using the linear animal model were also transformed to the liability scale using the standard transformation [19]. Genetic correlations between bTB environments were estimated using a series of bivariate sire linear models to determine whether bTB in the different environments was genetically the same trait. For the linear models, a likelihood ratio test was performed to investigate the existence of GxE by comparing the unconstrained bivariate models to a bivariate analysis where the genetic correlation between environments was constrained to one. A separate likelihood ratio test was performed for the linear models to investigate the existence of re-scaling effects between environments by comparing each bivariate model to a bivariate analysis where the variances in both environments were constrained to be equal.

## Results

The mean prevalence of bTB infection in the entire dataset (cows, heifers and steers) was 0.11 (Table 1). Prevalence of bTB in dairy and beef herds as well as in heifers, steers and cows is summarised in Table 1. The greatest prevalence of bTB infection was in cows, with a slightly greater prevalence in beef than in dairy cows. Prevalence of bTB infection was lower in heifers than in either cows or steers and was lowest in heifers from beef herds.

**Table 1:** **Results and summary statistics for univariate analyses for susceptibility to bovine tuberculosis (** ***M. bovis*** **) infection**

**Model**	**N**	**Mean**	**σ**_**g**_	**Heterosis (se)**	**Recombination (se)**	**h** ^**2**^ **(se)**
All herds	184 302	0.11	0.094	−0.002 (0.004)	−0.011 (0.011)*	0.11 (0.006)
Beef herds	32 191	0.14	0.114	−0.026 (0.007)*	−0.092 (0.027)*	0.13 (0.016)
Dairy herds	126 646	0.09	0.098	−0.032 (0.005)*	−0.031 (0.012)*	0.12 (0.007)
All cows	105 526	0.20	0.110	−0.026(0.005)*	−0.002 (0.014)	0.14 (0.009)
Cows in beef herds	10 202	0.22	0.108	0.004(0.016)	−0.003 (0.062)	0.10 (0.027)
Cows in dairy herds	87 918	0.19	0.109	−0.025(0.006)*	−0.009(0.015)	0.15 (0.010)
All heifers	56 904	0.09	0.098	0.003 (0.006)	−0.018 (0.018)*	0.15 (0.014)
Heifers in beef herds	13 468	0.10	0.124	−0.002 (0.011)	0.018 (0.037)	0.19 (0.030)
Heifers in dairy herds	33 987	0.07	0.068	−0.012 (0.009)*	−0.019 (0.019)*	0.08 (0.013)
All steers	21 872	0.10	0.102	0.000 (0.009)	−0.042 (0.032)*	0.15 (0.030)
Steers in beef herds	8610	0.11	0.119	−0.009 (0.012)	−0.044 (0.044)*	0.18 (0.040)
Steers in dairy herds	4741	0.09	0.095	−0.000 (0.018)	−0.032 (0.046)	0.17 (0.047)

Number of records (N); mean prevalence of infection (Mean); estimates of the genetic standard deviation (σ_g_); heterosis, recombination loss effects, and heritability (h^2^) for bTB susceptibility in the different populations; *denotes significance (P <0.05) from 0.

### Variance components

The estimate of heritability of susceptibility to *M. bovis* infection for the entire dataset, using a linear model, was 0.11 (Table 1). The estimate of heritability was greatest in heifers from beef herds (0.19) and lowest (0.08) in heifers from dairy herds (Table 1). Estimates of heritability of susceptibility to *M. bovis* infection were similar for dairy and beef herds. Younger animals (i.e., both heifers and steers) had greater heritability estimates than cows. The genetic standard deviation (SD) for susceptibility to *M. bovis* infection, when estimated across all animals, was 0.094 and was greatest in heifers from beef herds and lowest in heifers from dairy herds (Table 1). The estimate of genetic variance for bTB susceptibility was greater in beef herds than in dairy herds (Table 1) and was also greater (P <0.001) in cows than in younger animals (i.e., heifers and steers) but did not differ between heifers and steers. Increased breed heterozygosity was associated with lower susceptibility to bTB (Table 1); for example, 100% heterozygosity (i.e., a F1 crossbred) in dairy herds was associated with a 3.2% reduction in bTB prevalence. Estimates of recombination loss effects were different from zero in all sub-populations, except for the cow sub-populations, heifers in beef herds, and steers in dairy herds.

Estimates of the genetic correlation for bTB susceptibility was 0.55 between cows and heifers (standard error (se) = 0.048), 0.10 between cows and steers (se = 0.104), and 0.64 between steers and heifers (se = 0.082). The likelihood ratio test between nested models revealed that all genetic correlation estimates were significantly different from one (P <0.001).

**Figure 1:**
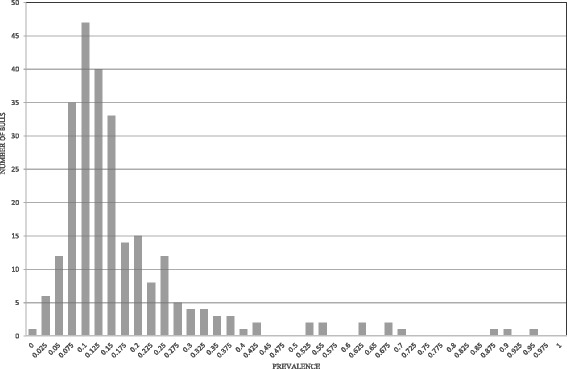
**Distribution of mean bTB prevalence in daughters of sires with bTB data.**

**Figure 2:**
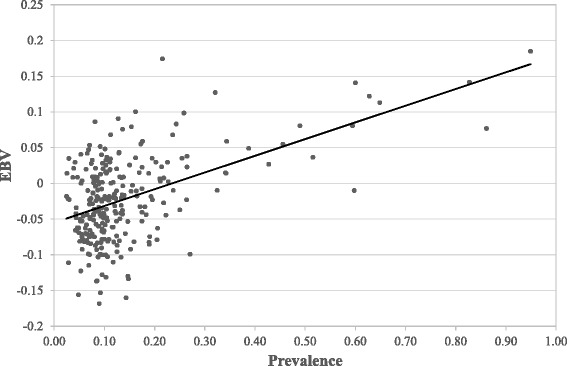
**Relationship of sire estimated breeding values (EBV) and mean prevalence of bTB infection (Prevalence).**

Figure 1 shows the mean bTB prevalence for daughters of sires with more than 50 female progeny in more than ten herds in the population under investigation. The mean bTB prevalence for daughters per sire ranged from 0.00 to 0.95. Figure 2 shows the mean bTB prevalence plotted against the EBV for bTB susceptibility for sires with more than 50 female progeny in more than ten herds; the correlation was 0.52.

### Breed effects

From the linear model using the full dataset, Holstein-Friesian sires had the lowest mean EBV compared to all other breeds (for sires born from 1992 onwards; Table 2), which indicates a reduced susceptibility to bTB. Simmental and Charolais sires had the greatest mean EBV (i.e. most susceptible to bTB infection) compared to all other breeds. The average EBV (weighted against the number of sires in each breed against the total number of sires across all breeds) in all beef breeds was 0.025 when considering sires with more than ten daughters; this was greater than the weighted average of −0.012 for all dairy breeds when considering only sires with more than ten daughters.

**Table 2:** Per breed mean estimated breeding values for susceptibility to bovine tuberculosis infection*

	**All sires**		**Sires with ≥10 progeny**	
**Breed**	**N**	**EBV (se)**	**N**	**EBV(se)**
Aberdeen Angus	379	0.005 (0.037)^D^	27	0.005 (0.050)^D^
Belgian Blue	102	0.042 (0.029)^B^	11	0.047 (0.054)^BAC^
Charolais	513	0.049 (0.034)^A^	48	0.050 (0.044)^BA^
Hereford	257	0.017 (0.028)^C^	11	0.017 (0.038)^BDC^
Holstein-Friesian	4977	−0.008 (0.045)^E^	959	−0.013 (0.0051)^E^
Jersey	84	0.015 (0.028)^C^	21	0.021 (0.036)^BAC^
Limousin	536	0.016 (0.035)^C^	50	0.010 (0.059)^DC^
Simmental	245	0.052 (0.033)^A^	16	0.053 (0.043)^A^

*For all sires and sires with more than 10 progeny; number of sires (N); mean estimated breeding value (EBV; standard error (se) in parentheses)^1^; ^1^EBV with the same superscripted letter are not significantly different from each other.

**Figure 3:**
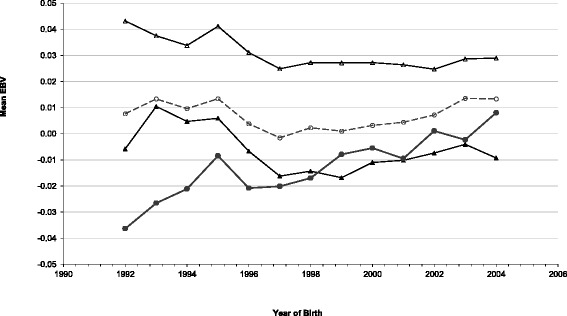
**Mean estimated breeding values (EBV) for bTB susceptibility against year of birth.** All sires (clear dots), sires with more than 10 daughters (solid dots), dairy breeds (solid triangles) and beef breeds (clear triangles).

Genetic trends for mean EBV of sires born between 1992 and 2004 are in Figure 3. Since 1992, the mean EBV was greater in beef sires than in dairy sires. The genetic susceptibility to bTB infection in beef and dairy animals has increased since 1999 but only the increase in dairy breeds was significantly different from zero (P <0.05).

### Genotype by environment effects

Summary statistics for bTB susceptibility across different bTB prevalence categories using a linear and threshold animal model are in Table 3. Using a linear animal model, the estimate of heritability of susceptibility to *M. bovis* infection was greatest for animals in herd-episodes of very high prevalence of bTB infection (>0.75 prevalence) and lowest for those in herd-episodes of very low prevalence (<0.25 prevalence). Using a threshold animal model, the estimate of heritability was lowest for animals in herd-episodes of very low prevalence of bTB infection (<0.25 prevalence) and greatest for those in herd-episodes of very high prevalence (>0.75). The genetic SD for bTB susceptibility, estimated using the linear animal model, was lowest for the extreme bTB prevalence categories, with the genetic SD of very low (<0.25 prevalence) and very high (>0.75 prevalence) prevalence categories being approximately equal to half that of the low (0.25 to 0.5 prevalence) and high (0.5 to 0.75 prevalence) prevalence categories (Table 3). The genetic SD for bTB susceptibility, estimated using the threshold animal model, increased greatly when the prevalence of bTB infection was greater than 0.75. After transforming heritability estimates from the linear model to the liability scale, heritability estimates increased as the prevalence of bTB infection increased from very low (<0.25) to very high (>0.75) prevalence, with a pronounced increase in heritability estimates in herd-episodes with a high prevalence (0.50 to 0.75) and a very high (>0.75) prevalence.

**Table 3:** **Results and summary statistics for univariate analyses for susceptibility to bovine tuberculosis infection across disease prevalences**

	**Very low**	**Low**	**High**	**Very high**
N	87 609	16 812	3082	3359
Mean	0.07	0.34	0.62	0.93
σ_g_ (LAM)	0.07^A^	0.16^B^	0.12^A^	0.09^C^
Heritability (LAM)	0.07 (0.008)	0.16 (0.024)	0.08 (0.048)	0.25 (0.072)
σ_g_ (TAM)	0.71	0.89	0.83	1.39
Heritability (TAM)	0.13 (0.012)	0.19 (0.016)	0.17 (0.037)	0.37 (0.081)
Transformed	0.25	0.27	0.31	0.88

Number of records (N); mean prevalence (Mean); estimates of the genetic standard deviation (σ_g_^1^); estimates of the heritability from the linear animal model (LAM), threshold animal model (TAM) and based on the standard Robertson transformation (Transformed), for environments with very low (<0.25), low (0.25 to 0.5), high (0.5 to 0.75) and very high (>0.75) prevalence of bTB infection; genetic variance estimates with the same superscripted letter were not different (P <0.05) from each other.

Estimates of genetic correlations between susceptibility to bTB infection in different environments were all positive (Table 4) and ranged from 0.06 (environments with low and very high bTB prevalence) to 0.86 (environments with low and very low bTB prevalence). All genetic correlation estimates were less than one (P <0.05) and estimates of the genetic variance in different environments also differed from each other (P <0.05) with the exception of the very low (<0.25) and high (0.5 to 0.75) bTB prevalence environments (Table 3).

**Table 4:** **Estimates of genetic correlations** ^**1**^ **for susceptibility to bTB infection between environments of differing disease prevalence**

	**Very low**	**Low**	**High**
Low	0.86 (0.043)		
High	0.65 (0.114)	0.84 (0.133)	
Very high	0.08 (0.107)	0.06 (0.147)	0.31(0.253)

Very low (<0.25), low (0.25 to 0.5), high (0.5 to 0.75) and very high (>0.75) bTB prevalence; all genetic correlations differed (P <0.05) from 1.

## Discussion

The contribution of breeding programs to phenotypic gains in animal performance has been well documented [20,21]. Hence, a breeding program aimed at improving resistance to bTB can form an integral component of a national eradication program if heritable variation in bTB exists and can be exploited. The economic importance of bTB in Ireland is well established [7] and the centrally stored phenotypic data on bTB status at the individual level could be used to estimate breeding values. Therefore, the objective of our study was to quantify the genetic variation of the susceptibility to bTB in cattle. A novel component of this study was the estimation of genetic variation for bTB susceptibility in beef cattle.

### Variance components

In this study on dairy and beef cattle, a heritability of 0.11 for susceptibility to bTB was estimated using a linear model, which is between documented estimates of most production diseases (<0.05; [21]) and animal performance traits (~0.30; [22-24]). The heritability estimates of susceptibility to bTB reported in the present study, especially those for dairy cows, are consistent with previous linear model estimates for both Irish dairy [12] and UK dairy [13] cows. Moreover, the reported heritability estimates of bTB, including those from the present study, are similar to those documented for paratuberculosis (i.e., *Mycobacterium avium*) [25-27], which shares some epidemiological similarities with bTB [28]. Furthermore, our heritability estimates of susceptibility to bTB using a linear model did not vary considerably across the populations investigated (beef, dairy, cows, heifers, steers); differences in estimates were due to a combination of differences in residual and genetic variances between populations.

Despite the similarities in heritability estimates among the different studies, the genetic variance estimates for susceptibility to bTB reported in the present study were two to three times greater than those documented by Bermingham et al. [12] in a sub-population of the animals used in the present study (i.e. that study was limited to dairy cows and heifers). The greater genetic variance reported in the present study could be due to the greater diversity of breeds included. Our estimates of genetic variance for susceptibility to bTB were also three to six times greater than those estimated for Johne’s disease in a group of 4603 US Holstein-Friesian cows [26], which was also treated as a binary trait and estimated using a linear model.

### Breed effects

Previously reported data on between-breed differences in susceptibility to bTB corroborate our results. For example, Ameni et al. [29] reported a greater susceptibility and severity in the pathology of bTB in Holsteins compared to East African Zebu and Zebu-Holstein crossbreds. Other studies have also documented significant breed differences for many health traits, such as somatic cell score [30], lameness [31,32], and bovine respiratory disease [33].

The observed recent genetic trends in susceptibility to bTB (Figure 3) suggest that, although the breeding goal for Irish dairy cattle is to select for longevity, fertility, and milk production [34], genetic merit for resistance to bTB appears to be deteriorating in dairy cattle. Although the estimate of the genetic trend for susceptibility to bTB in beef cattle was not different from zero, genetic resistance to bTB has not improved since the 1990s (Figure 3). It is therefore important to consider including resistance to bTB in the Irish national breeding goals, especially for dairy cows. Another recommendation is that other countries also evaluate genetic trends for susceptibility to bTB in their cattle populations and, where necessary, take remedial action. The contribution of breeding programs to improving genetic merit for animal health traits has been clearly documented in Scandinavian dairy cow populations [35,36]. In countries like Ireland, where routine testing of animals for bTB status is undertaken, it should be possible to generate accurate genetic evaluations for bTB to inform better selection decisions. Some fears have been expressed that breeding for disease resistance will lead to (or otherwise be associated with) reduced responsiveness to the SICTT. This concern was considered in detail by Bishop and Woolliams [37] and they concluded there was no evidence that such a scenario would arise.

### Genotype by environment effects

The genetic variance of susceptibility to bTB in environments with different bTB prevalence levels has not previously been documented. The pattern of genetic variances of susceptibility to bTB in environments with different bTB prevalence levels, estimated using an animal linear mixed model, was similar to the trend in genetic variances estimated for somatic cell score in environments with different herd average somatic cell scores [38] (i.e. it was least in the most extreme environments). However, the genetic variance pattern observed in the present study was in direct contrast to the pattern of genetic variance estimates reported for Johne’s disease in Dutch Holstein-Friesian cows [39], which increased with herd prevalence for Johne’s disease. Nonetheless, the pattern in genetic variance for susceptibility to bTB when estimated with a threshold animal model in the present study was similar to that observed for Johne’s disease [39] and herd prevalence (Table 3). Estimates of variance components in the different prevalence groups were similar to those reported by van Hulzen et al. [39], using a linear model, with the exception of the very high prevalence environment. An inverse relationship existed between estimates of genetic variance and heritability using the linear model across the different prevalence groups, while one would expect a positive relationship [37]. This could be due to the fewer animals in the more extreme environmental categories or selection/culling. To ensure that there was no impact of the estimation algorithm on estimates of the variance components, the zeros and ones in the high prevalence environment were switched and variance components re-estimated using the linear model; this did not impact the estimated variance components. Although heritability estimates differed between the threshold animal model and the linear model estimates transformed to the liability scale, the heritability of susceptibility to bTB in the very high prevalence herds (>0.75) was always greatest, regardless of the model used. Nonetheless, caution should be taken in interpreting the large heritability in the very high prevalence environment due to the smaller number of records.

The estimates of the genetic correlation of susceptibility to bTB between cows and heifers in the present study (0.55) was similar to the estimate of 0.53 documented by Bermingham et al. [12] comparing also cows and heifers. While differences in genetic variance for disease susceptibility between females and males have been documented (e.g., for tick infection in Australian beef cattle [40]), there is little information on genetic correlations for disease susceptibility between sexes. Nonetheless, estimates of genetic correlations for performance traits in different sexes have been reported. For example, Pabiou et al. [24] reported genetic correlations that ranged from 0.54 to 0.81 for wholesale carcass cut traits in steers and heifers, while Stalhammar and Philipsson [41] documented genetic correlations for post-weaning and weanling gain in male and female Swedish beef cattle that ranged from 0.4 to 1.0. Moreover, genetic correlations between the same performance traits for animals of different ages have also been reported; for example, genetic correlations of animal weight at weaning, post-weaning weight and weight of cows have been reported to range from 0.16 to 0.79 [42]. These results corroborate estimates of genetic correlations for susceptibility to bTB being less than one as documented in the present study. These less than one genetic correlations between animal types, indicates that the bTB phenotype is possibly under different genetic control in the cows, heifers, and steers.

Estimates of genetic correlations of susceptibility to bTB between environments with different bTB prevalence levels were weakest for the environment with the greatest pathogen load (i.e., >0.75 bTB prevalence). This suggests that the genetic background of susceptibility to bTB in environments under a very high pathogen load is different to that in environments under a lower pathogen load. Calus et al. [43] observed a similar phenomenon when they investigated the genetics of somatic cell score in Dutch Holstein cows across environments (i.e., herds) based on different mean bulk tank somatic cell score. Furthermore, Calus et al. [43] reported the weakest genetic correlations with environments exhibiting the greatest somatic cell count. The weak genetic correlations for bTB observed in the present study with environments with the greatest prevalence of bTB could be due to the extremely high pathogen load suggesting possibly a different underlying genetic or biological systems (e.g., innate, adaptive) contributing to whether or not the animal becomes infected with bTB. Another explanation could be the high likelihood that all animals in such an environment are exposed to the pathogen, and thus the susceptibility to bTB in this environment, as defined in the present study, may better reflect the true estimate of bTB susceptibility. The genetic variance of susceptibility to bTB was also least in the environment with the greatest bTB prevalence, but the heritability of bTB in the very high prevalence environment was greatest. The high pathogen load environment is somewhat analogous to experimental inoculation with bTB and the high heritability for bTB in this environment in the present study (0.25) is closer to the heritability of 0.48 reported for deer experimentally infected with *M. bovis* (0.48; [44]). It must be noted however, that this heritability estimate of susceptibility to bTB in deer is considered to be high due to the naivety of the deer to TB. Despite this, it could be suggested that the heritability of susceptibility to bTB may actually be greater than the value of 0.11 reported in the present study across all data once the assumption that all animals are equally exposed to the pathogen is fulfilled.

### Methodological strengths and weaknesses

A number of factors that contribute to the under-estimation of the heritability of disease susceptibility in livestock have been documented [37] using simulated data. Bishop and Woolliams [37] identified three factors which contribute to the underestimation of the heritability of disease susceptibility in livestock:

1. Incomplete exposure of animals to the pathogen, resulting in some animals not having the opportunity to express their genotype for the disease. The degree of underestimation is linear with the decreased probability of complete exposure. The strict inclusion criteria for herd-episodes imposed in the present study aimed at maximising the probability of complete exposure. However, complete exposure may not have been reached in some cases. With the observed prevalence of bTB of 0.11 in the entire dataset (Table 1), according to Bishop and Woolliams [37] equations, an exposure probability of 50% would have been needed in the present study to estimate a sufficiently accurate heritability of susceptibility to bTB.
2. Incomplete sensitivity and specificity of the diagnostic tests used to classify animals as healthy or diseased. Bermingham et al. [45] illustrated that the effects of imperfect SICTT sensitivity resulted in an underestimation of the heritability of susceptibility to bTB in Irish and UK dairy cows. Furthermore, Bishop and Woolliams [37] concluded that imperfect sensitivity to SICTT has a large effect on the estimate of heritability of susceptibility to bTB, especially when the disease prevalence is as low as 0.10, similar to that observed in the present study. It is also well-documented that the standard SICTT can detect only between 40 and 80% of infected animals [46,47]. In the present study, to account for the sensitivity of SICTT, only the herd-episodes for which 20% of ‘reactors’ presented bTB lesions were included, which implies that bTB infection was reasonably established in these herd-episodes.
3. Misclassification of animals due to the dynamic expression of the disease over the course of an infection. With bTB infection, many animals will not present the infection for some time [15] and may therefore not be identified when testing is undertaken. Furthermore, during the period of bTB infection, animals within a herd will be at different stages of infection. Animals at the earlier stages of infection will be difficult to detect (since sensitivity to testing is low), while animals at later stages will be easier to detect. This misclassification will cause underestimation of the heritability, analogous to the effect of imperfect test sensitivity. To reduce the probability of misclassification because of dynamic expression, a number of standard testing procedures for bTB are carried out by the Irish Department of Agriculture, Food and Marine, including annual testing of all animals in herds and continuous surveillance of animals at slaughter. Re-testing of herds after the identification of a ‘standard reactor’ or an ‘inconclusive reactor’, at two-month intervals also aims at reducing the likelihood of misclassifying animals due to this effect.

## Conclusions

For a trait to be included in a breeding goal it must have some relevance (e.g., economical or social), be measurable or correlated with a measureable phenotype, and exhibit genetic variation. Bovine tuberculosis is of economic importance [3,7], which justifies its consideration for inclusion in the breeding goal. The annual testing of whole herds for bTB and the storage of these data in a national database, as well as access to pedigree information, animal movements and other systematic environmental effects pertaining to the test (e.g., herd, season, age of animal), imply that routine genetic evaluations are possible for bTB if genetic variation in susceptibility to bTB exists. Results from this study clearly show that exploitable genetic variation in susceptibility to bTB exists. Moreover, the current genetic trend for sires with more than 50 progeny with bTB data in the national population suggests that susceptibility to bTB infection in the progeny of these animals may be increasing. All these points constitute a strong argument to consider increased resistance to bTB as a trait for selection in Irish dairy and beef breeding and part of the national strategy for bTB eradication in cattle. Moreover, its usefulness in breeding strategies for other cattle populations that are not free of bTB should be investigated. While fears have been expressed that selection for bTB resistance will reduce sensitivity to SICTT testing, it is thought that such a scenario will not arise. Although some estimates of the genetic correlation of susceptibility to bTB between sub-populations were less than 0.8, which means that it cannot be considered as the same trait across these sub-populations, performing separate genetic evaluations for each animal type or for different environments in a multi-trait genetic evaluation may be impractical. Moreover, since all genetic correlation estimates between the different sub-populations were positive, a genetic evaluation using all animal types and environments combined is expected to improve genetic merit across all sub-populations.

## References

[1] Garnier T, Eiglmeier K, Camus JC, Medina N, Mansoor H, Pryor M, Duthoy S, Grondin S, Lacroix C, Monsempe C, Simon S, Harris B, Atkin R, Doggett J, Mayes R, Keating L, Wheeler PR, Parkhill J, Barrell BG, Cole ST, Gordon SV, Hewinson RG (2003). The complete genome sequence of *Mycobacterium bovis*. Proc Natl Acad Sci U S A.

[2] Abernethy DA, Upton P, Higgins IM, McGrath G, Goodchild AV, Rolfe SJ, Broughan JM, Downs SH, Clifton-Hadley R, Menzies FD, de la Rua-Domenech R, Blissitt MJ, Duignan A, More SJ (2013). **Bovine tuberculosis trends in the UK and the Republic of Ireland, 1995**–**2010**. Vet Rec.

[3] Perry BD, Randolph TF, McDermott JJ, Sones KR Thornton PK (2002). Investing in animal health research to alleviate poverty.

[4] Allen AR, Minozzi G, Glass EJ, Skuce RA, McDowell SW, Woolliams JA, Bishop SC (2010). Bovine tuberculosis: the genetic basis of host susceptibility. Proc Biol Sci.

[5] More SJ, Good M (2006). The tuberculosis eradication programme in Ireland: a review of scientific and policy advances since 1988. Vet Microbiol.

[6] More SJ (2009). What is needed to eradicate bovine tuberculosis successfully: An Ireland perspective. Vet J.

[7] Good M (2006). Bovine tuberculosis eradication in Ireland. Irish Vet J.

[8] Payne A, Boschiroli ML, Gueneau E, Moyen JL, Rambaud T, Dufour B, Gilot-Fromont E, Hars J (2013). Bovine tuberculosis in “Eurasian” badgers (*Meles meles*) in France. Eur J Wildl Res.

[9] Zanella G, Bar-Hen A, Boschiroli ML, Hars J, Moutou F, Garin-Bastuji B, Durand B (2012). Modelling transmission of bovine tuberculosis in Red deer and Wild boar in Normandy, France. Zoonoses Public Health.

[10] Aranaz A, de Juan L, Montero N, Sanchez C, Galka M, Delso C, Alvarez J, Romero B, Bezos J, Vela AI, Briones V, Mateos A, Dominguez L (2004). Bovine tuberculosis (Mycobacterium bovis) in wildlife in Spain. J Clin Microbiol.

[11] Schoning JM, Cerny N, Prohaska S, Wittenbrink MM, Smith NH, Bloemberg G, Pewsner M, Schiller I, Origgi FC, Ryser-Degiorgis MP (2013). Surveillance of bovine tuberculosis and risk estimation of a future reservoir formation in wildlife in Switzerland and Liechtenstein. PLoS ONE.

[12] Bermingham ML, More SJ, Good M, Cromie AR, Higgins IM, Brotherstone S, Berry DP (2009). Genetics of tuberculosis in Irish Holstein-Friesian dairy herds. J Dairy Sci.

[13] Brotherstone S, White IM, Coffey M, Downs SH, Mitchell AP, Clifton-Hadley RS, More SJ, Good M, Woolliams JA (2010). Evidence of genetic resistance of cattle to infection with Mycobacterium bovis. J Dairy Sci.

[14] Monaghan ML, Doherty ML, Collins JD, Kazda JF, Quinn PJ (1994). The tuberculin test. Vet Microbiol.

[15] de la Rua-Domenech R, Goodchild AT, Vordermeier HM, Hewinson RG, Christiansen KH, Clifton-Hadley RS (2006). Ante mortem diagnosis of tuberculosis in cattle: a review of the tuberculin tests, gamma-interferon assay and other ancillary diagnostic techniques. Res Vet Sci.

[16] O'Keeffe JJ, Crowley MJ (1995). Episode classification in the eradication of bovine tuberculosis: a different perspective. Tuberculosis Investigation Unit, Dublin. Irish Department of Agriculture Food and Marine.

[17] VanRaden PM, Sanders AH (2003). Economic merit of crossbred and purebred US dairy cattle. J Dairy Sci.

[18] Gilmour AR, Gogel BJ, Cuillis BR, Thompson R (2009). ASReml User Guide Release 3.0.

[19] Dempster ER, Lerner IM (1950). Heritability of threshold characters. Genetics.

[20] Berry DP (2008). Genetics - A tool to improve productivity and profitability. Int J Dairy Technol.

[21] Berry DP, Bermingham ML, Good M, More SJ (2011). Genetics of animal health and disease in cattle. Irish Vet J.

[22] Berry DP, Buckley F, Dillon P, Evans RD, Rath M, Veerkamp RF (2003). Genetic parameters for body condition score, body weight, milk yield, and fertility estimated using random regression models. J Dairy Sci.

[23] McHugh N, Evans RD, Amer PR, Fahey AG, Berry DP (2011). Genetic parameters for cattle price and body weight from routinely collected data at livestock auctions and commercial farms. J Anim Sci.

[24] Pabiou T, Fikse WF, Nasholm A, Cromie AR, Drennan MJ, Keane MG, Berry DP (2009). Genetic parameters for carcass cut weight in Irish beef cattle. J Anim Sci.

[25] Berry DP, Good M, Mullowney P, Cromie AR, More SJ (2010). Genetic variation in serological response to Mycobacterium avium subspecies paratuberculosis and its association with performance in Irish Holstein-Friesian dairy cows. Livest Sci.

[26] Gonda MG, Chang YM, Shook GE, Collins MT, Kirkpatrick BW (2006). Genetic variation of Mycobacterium avium ssp paratuberculosis infection in US Holsteins. J Dairy Sci.

[27] Mortensen H, Nielsen SS, Berg P (2004). Genetic variation and heritability of the antibody response to Mycobacterium avium subspecies paratuberculosis in Danish Holstein cows. J Dairy Sci.

[28] Sweeney RW (1996). Transmission of paratuberculosis. Vet Clin North Am Food Anim Pract.

[29] Ameni G, Aseffa A, Engers H, Young D, Gordon S, Hewinson G, Vordermeier M (2007). High prevalence and increased severity of pathology of bovine tuberculosis in Holsteins compared to zebu breeds under field cattle husbandry in central Ethiopia. Clin Vaccine Immunol.

[30] Schutz MM, Vanraden PM, Wiggans GR (1994). Genetic variation in lactation means of somatic-cell scores for six breeds of Dairy cattle. J Dairy Sci.

[31] Baird LG, O'Connell NE, McCoy MA, Keady TWJ, Kilpatrick DJ (2009). Effects of breed and production system on lameness parameters in dairy cattle. J Dairy Sci.

[32] Mattiello S, Battini M, Andreoli E, Barbieri S (2011). Short communication: Breed differences affecting dairy cattle welfare in traditional alpine tie-stall husbandry systems. J Dairy Sci.

[33] Snowder GD, Van Vleck LD, Cundiff LV, Bennett GL (2005). Influence of breed, heterozygosity, and disease incidence on estimates of variance components of respiratory disease in preweaned beef calves. J Anim Sci.

[34] Berry DP, Lee JM, Macdonald KA, Stafford K, Matthews L, Roche JR (2007). Associations among body condition score, body weight, somatic cell count, and clinical mastitis in seasonally calving dairy cattle. J Dairy Sci.

[35] Heringstad B, Klemetsdal G, Ruane J (2000). Selection for mastitis resistance in dairy cattle: a review with focus on the situation in the Nordic countries. Livest Prod Sci.

[36] Heringstad B, Klemetsdal G, Ruane J (2001). Responses to selection against clinical mastitis in the Norwegian cattle population. Acta Agr Scand A Anim Sci.

[37] Bishop SC, Woolliams JA (2010). On the genetic interpretation of disease data. PLoS ONE.

[38] Banos G, Shook GE (1990). Genotype by environment interaction and genetic correlations among parities for somatic-cell count and milk yield. J Dairy Sci.

[39] van Hulzen KJ, Nielen M, Koets AP, de Jong G, van Arendonk JA, Heuven HC (2011). Effect of herd prevalence on heritability estimates of antibody response to Mycobacterium avium subspecies paratuberculosis. J Dairy Sci.

[40] Seifert GW (1971). Variations between and within breeds of cattle in resitance to field infestations of the cattle tick (*Boophilus microplus*). Aust J Agric Res.

[41] Stalhammar H, Philipsson J (1997). Sex-specific genetic parameters for weaning and post-weaning gain in Swedish beef cattle under field conditions. Acta Agr Scand A Anim Sci.

[42] Mc Hugh N, Evans RD, Amer PR, Fahey AG, Berry DP (2011). Genetic parameters for cattle price and body weight from routinely collected data at livestock auctions and commercial farms. J Anim Sci.

[43] Calus MPL, Janss LLG, Veerkamp RF (2006). Genotype by environment interaction for somatic cell score across bulk milk somatic cell count and days in milk. J Dairy Sci.

[44] Mackintosh CG, Qureshi T, Waldrup K, Labes RE, Dodds KG, Griffin JFT (2000). Genetic resistance to experimental infection with Mycobacterium bovis in red deer (*Cervus elaphus*). Infect Immun.

[45] Bermingham ML, Brotherstone S, Berry DP, More SJ, Good M, Cromie AR, White IM, Higgins IM, Coffey M, Downs SH, Glass EJ, Bishop SC, Mitchell AP, Clifton-Hadley RS, Woolliams JA (2011). Evidence for genetic variance in resistance to tuberculosis in Great Britain and Irish Holstein-Friesian populations. BMC Proc.

[46] Conlan AJK, McKinley TJ, Karolemeas K, Pollock EB, Goodchild AV, Mitchell AP, Birch CPD, Clifton-Hadley RS, Wood JLN (2012). Estimating the hidden burden of bovine tuberculosis in Great Britain. PLoS Comput Biol.

[47] Karolemeas K, de la Rua-Domenech R, Cooper R, Goodchild AV, Clifton-Hadley RS, Conlan A, Mitchell AP, Hewinson RG, Donnelly CA, Wood JL, McKinley TJ (2012). Estimation of the relative sensitivity of the comparative tuberculin skin test in tuberculous cattle herds subjected to depopulation. PLoS ONE.

